# Acceptance and adherence to non-invasive positive pressure ventilation in people with chronic obstructive pulmonary disease: a grounded theory study

**DOI:** 10.3389/fpsyg.2023.1134718

**Published:** 2023-08-03

**Authors:** Eleonora Volpato, Paolo Innocente Banfi, Francesco Pagnini

**Affiliations:** ^1^Department of Psychology, Università Cattolica del Sacro Cuore, Milan, Italy; ^2^IRCCS Fondazione Don Carlo Gnocchi, Milan, Italy

**Keywords:** chronic obstructive pulmonary disease (COPD), non-invasive positive pressure ventilation (NPPV), acceptance, adherence, constructivist grounded theory

## Abstract

**Introduction:**

Non-Invasive Positive Pressure Ventilation (NPPV) is an established treatment for people with Chronic Obstructive Pulmonary Disease (COPD), but it is often improperly used or rejected. The patterns of acceptance and adherence to NPPV, conceiving constraints, and strengths related to its adaptation have not been explored from a qualitative perspective yet.

**Objectives:**

This study aims to qualitatively explore patterns of adaptation to NPPV in people affected by COPD and to identify the core characteristics and the specific adaptive challenges during the adaptation process.

**Methods:**

Forty-two people with moderate or severe COPD were recruited and 336 unstructured interviews were conducted. A Constructivist Grounded Theory was used to gather and analyze data: the transcriptions were mutually gathered in open, selective, and theoretical phases, with open, selective, and theoretical coding, respectively.

**Results:**

The analysis resulted in a non-linear and dynamic process, characterized by three phases: deciding, trying NPPV, and using NPPV. The patterns revealed that positive and negative NPPV experiences, together with beliefs, emotions, stressful mental states, and behaviors result in different acceptance and adherence rates.

**Discussions:**

These findings may be helpful to implement new care strategies to promote acceptance and adherence to NPPV.

## 1. Background

Chronic obstructive pulmonary disease (COPD) is the third leading cause of death worldwide, causing 3.23 million deaths in 2019 (World Health Organization, 2014; Venkatesan, 2022). COPD is a common, preventable, treatable, and progressive chronic lung disease characterized by airflow limitation that is not fully reversible. Progressive dyspnea, chronic cough, and/or sputum production are considered the main symptoms, and a history of exposure to risk factors (i.e., smoke, air pollution, exposure to particles) is also a major consideration (Vogelmeier et al., 2017). Disease progression often leads to severe hypoxemia (Lacasse et al., 2022), resulting in daytime fatigue (Ebadi et al., 2021), sleepiness (Li et al., 2021), reduced exercise tolerance, impaired cognitive functions (Simargi et al., 2022; Zhao and Zhou, 2022), anxiety (Martínez-Gestoso et al., 2022), and depression (Peiffer et al., 2021; Martínez-Gestoso et al., 2022). Long-term Oxygen Therapy (LTOT) has been found to improve the quality of life (Pavlov et al., 2018). Another respiratory treatment is Non-Invasive Positive Pressure Ventilation (NPPV), which refers to the administration of synchronized ventilatory support without using an invasive artificial airway (endotracheal tube or tracheostomy tube). NPPV is considered standard therapy for patients with Acute Respiratory Failure (ARF) or Chronic Respiratory Failure (CRF) due to COPD. Improvements in both survival and intubation rates are well-rooted (Windisch et al., 2002; Roberts et al., 2008; Comellini et al., 2019), reducing mortality and lowering hospital care costs (Abubacker et al., 2021; Burns et al., 2022). NPPV helps to relieve symptoms such as daytime fatigue, and dyspnea, normalizing CO_2_ and O_2_ levels in the body; it can be effective in reducing time in hospital and preventing exacerbations (Comellini et al., 2019; Wilson et al., 2020). In interventions such as NPPV, balancing the comfort of the patient and the parameters setting of ventilatory support is a recognized part of patient-centered care and it is more and more desirable to increase both compliance and adherence (Barry and Edgman-Levitan, 2012). NPPV’s rejection or improper use represents big challenges and has been associated with baseline dyspnea, acute acidosis, and intolerance to the treatment, in particular to the mask (Hess, 2011), air leaks (Tan et al., 2022), and the size of the dead space (Elliott, 2004).

Previous studies have used quantitative research designs and have paid attention to the influence of NPPV from a medical perspective, resulting in less literature about the lived experiences and the process of adaptation to NPPV’s usage (Ngandu et al., 2016; Dennis et al., 2022; McCormick et al., 2022). These few studies noted that COPD people expressed initial feelings of being trapped (Iosifyan et al., 2019) and fears that they will become completely dependent on others (Torheim and Gjengedal, 2010; Beckert et al., 2020) or on medical devices (Lindahl et al., 2005), and experienced anxiety or discomfort (Christensen et al., 2018). However, only one study tried to develop a behavioral model of their experience with NPPV, showing that, after both a restrained and a transition phase, the patient tends to develop tolerance or total rejection towards NPPV (Sørensen et al., 2014). Furthermore, to our knowledge, no study has explored the perspective of COPD patients on adaptation to NPPV not only considering the first hours of adaptation but rather the first weeks, with a constructivist approach based on grounded theory.

### 1.1. Objectives

The main aim of this study was to develop a theoretical account of the pattern of acceptance and adherence to NPPV in COPD people, conceptualizing barriers and facilitators that may explain variations during the adaptation process. In particular, the aim was to identify the characteristics and management levers of NPPV in people affected by COPD and suggestions to guide its adaptation and adherence. The research questions orienting this study were “What is the main concern during the adaptation to NPPV for COPD patients?” and “How did the COPD patients cope with NPPV?”

## 2. Methods

### 2.1. Ethical approval and informed consent

This study was approved by the Ethics Committee of the IRCCS Fondazione Don Carlo Gnocchi (reference: 15 February 2015), in Milan (Italy), in the mainframe of a large RCT on acceptance and adherence to NIV in people with COPD (ref. ClinicalTrials.gov ID: NCT02499653).

All the participants signed a Consent Form after being informed verbally and in writing about the study, their right to withdraw at any time, anonymity, and confidentiality. We confirm that all personal identifiers have been removed or disguised so the individuals described are not identifiable and cannot be identified through the details of the story.

### 2.2. Design

We used a qualitative approach, according to the Constructivist Grounded Theory (CGT), which favors the generation of a conceptual understanding from a bottom-up standpoint of textual data (Glaser, 2002; Charmaz, 2006, 2014; Glaser and Strauss, 2017). CGT allows emphasizing both conceptualization and the interactive process in an area that is not extensively evaluated. Moreover, the constructivist aspect of the method allowed for the elaboration of the theoretical coding session by session, resulting in a dynamic deliberation of the researchers (Charmaz, 2006, 2014). The research strategy followed the grounded theory method as presented by Glaser and Strauss (2017), developed by Glaser (1978), and influenced by Strauss and Corbin (1998) as described in Hylander (2003). This study followed the COREQ (COnsolidated criteria for REporting Qualitative research) (Tong et al., 2007).

### 2.3. Participant selection

#### 2.3.1. Sampling and method of approach

Eligible participants were approached face-to-face at the Cardio-Respiratory Rehabilitation Unit of IRCCS Fondazione Don Carlo Gnocchi, Milan (Italy), after the respiratory visit with the Pulmonologist. They were recruited according to purposive sampling, respecting the inclusion and exclusion criteria described in paragraph 2.3.2, and proceeded until theoretical saturation was reached (i.e., the emergent theory was fully represented by the data collected). In this regard, of the relatively homogenous population and the lack of a previous theoretical model about the adaptation to NPPV, analysis was conducted after every 15 interviews, and data saturation was determined when no new codes, themes, or patterns emerged (Fusch and Ness, 2015).

#### 2.3.2. Inclusion and exclusion criteria

Participants were included if they had a confirmed diagnosis of COPD according to Global Initiative for Chronic Obstructive Lung Disease (GOLD) criteria (Venkatesan, 2022), from moderate [Stage 2–50% ≤ Forced Expiratory Volume in 1 Second (FEV_1_) < 80% predicted] to severe (Stage 3– 30% ≤ FEV_1_ < 50% predicted) along with using NPPV.

Patients admitted with acute decompensated hypercapnic exacerbations of COPD requiring acute NPPV were screened for eligibility at least 2 weeks after the resolution of decompensated acidosis (arterial pH >7.30). Patients were required to have persistent hypercapnia (PaCO_2_ > 53 mm Hg) and hypoxemia (PaO_2_ < 60 mm Hg; or cor pulmonale; >30% of sleep time with oxygen saturation < 90% as measured by pulse oximetry); and arterial pH > 7.30 while breathing room air.

Exclusion criteria were symptoms indicating a severe cognitive and/or behavioral dysfunction [Mini-Mental State Examination (MMSE) corrected<21] (Folstein, 1983).

### 2.4. Data collection

#### 2.4.1. Instruments

Unstructured interviews were undertaken because we had established through a literature review that little was known about the nature of the characteristics and management levers of NPPV (Ngandu et al., 2016) and, in particular, the underline process of adaptation to NPPV in COPD (Sørensen et al., 2014). Moreover, our topic was broad, and we did not set out to focus on any factor of the adaptation process. A story-telling approach was used to encourage the participants to narrate their relationship with the first approach to NPPV. Hence, most clinical sessions began with the story of what was new for the participants: therefore, they mostly talked about their first approach to NPPV. Every time participants struggled with phrasing an episode, prompts were applied. An inductive interviewing approach encouraged subjects to express themselves freely, thereby allowing themes to emerge.

#### 2.4.2. Duration

Concurrent data collection and analysis, constant comparative analysis, and theoretical sampling were carried out between June 2015 and December 2018. The duration of each face-to-face conversation was about 30–45 min, and they were conducted during the adaptation process to NPPV weekly for a total of 4–8 sessions, according to the patient’s needs, and during a psychological support intervention for an average time of about 1–2 months and a half per participant. The clinical sessions were audiotaped and transcribed verbatim immediately after their conclusion. Moreover, some field notes and analytic memos were annotated both during and after the interview to aid further interpretation as well as for self-reflection purposes. Data collection analysis has been implemented in parallel to refine concepts and theory.

#### 2.4.3. Setting of data collection

Clinical sessions were conducted face-to-face by the first author (EV), a trained Psychologist, either in the participants’ homes or in a clinic room at the Cardio-Respiratory Rehabilitation Unit. There were no previous relationships between the study participants and the researcher before the start of the study, as new admissions at the mentioned department. No one else was present behind the participants and the researcher, to allow a comfortable environment and treat them as the main informants and competent commentators on their adaptation process to NPPV’s usage.

### 2.5. Data analysis

Constructivist Grounded Theory Approach was used for data analysis (Charmaz, 2006, 2014; Glaser and Strauss, 2017). This approach is based on the concept of emergent themes, which are not used only to explore an issue but also to construct a cohesive idea or theory about an investigated phenomenon. Following the criteria for methodological rigor in qualitative research (Creswell, 2014), the transcribed data were jointly collected in open, selective, and theoretical phases (Glaser and Strauss, 2017) until conceptual density was achieved. They were categorized using the constant comparison model. Firstly, each sentence was analyzed line by line to elicit the categories and define their meaning (open coding). Secondly, the relationship between categories was considered to allow the formulation of conceptual main classification (selective coding). Finally, the integration between conceptual categories was favored to create a wide theoretical model about the process of adaptation to NPPV (theoretical coding). The analysis was carried out using the NVivo software (QSR International®, version 11).

#### 2.5.1. Validity of the analysis

Data were validated by coming back to and scrutinizing all of them for meaning that had interfered. The grounded theory does not allow any sort of hypothesis. However, there is an assumption that might have influenced data collection, analysis, and interpretation. Indeed, a positive partnership with the patient could improve the adaptation to NPPV. To mitigate the impact of this assumption during the stages of the study, reflexive discussions among authors were conducted. Furthermore, coding and emergent findings were discussed regularly, to orient both subsequent sampling and analysis. Triangulation was sought with another psychologist (FP) and a clinician (PB) to enrich and fine-tune the analysis that converged on the final interpretation. All discrepancies have been discussed and a final consensus was reached.

## 3. Results

### 3.1. Characteristics of the study participants

According to CGT methodology, sampling evolved from a purposive to a theoretical strategy (Charmaz, 2006, 2014). The sampling strategy assured the gathering of relevant and diverse data inherent to the research question. Successively, to test stories about the process of successful adaptation or rejection to NPPV, we opted for theoretical sampling. The initial objective was to collect up to 30 sessions, but it was broadened to address the arising themes in the data. Therefore, a total of 336 sessions were included in the process of the analysis and reported the adaptation to NPPV of 42 participants (males = 19; females = 23; mean age = 77.02, SD = 7.53). Each session lasted a mean of 40 min (SD = 2.5) and 28.57% (*n* = 12) needed fewer than 8 sessions (a mean of 5 sessions over a total average period of 2 months).

None of the participants recruited withdrew from the study or was asked to interrupt the interviews.

The sociodemographic features of the sample are illustrated in Table 1.

**Table 1:** Socio-demographic characteristics of the overall sample.

Socio-demographic characteristics	
Variable	Sample (*n* = 42)
Gender, M/F (% male)	19/23 (45.2)
Age, mean (SD)	77.02 (7.53)
Marital status, *n* (%)	
Single	2 (4.8)
Married	29 (69)
Widower	7 (16.7)
Separated	3 (7.1)
Divorced	0
Without any family or social support	1 (2.4)
Cohabitants, *n* (%)	
Spouse	23 (54.8)
Mather	1 (2.4)
Father	0
Sons	4 (9.5)
Other relatives	2 (4.8)
Carer	1 (2.4)
Friends	0
Senior centre	0
Alone	6 (14.3)
Spouse and carer	1 (2.4)
Spouse and sons	3 (9.6)
Educational Level, *n* (%)	
None	4 (9.5)
Primary school	14 (33.3)
Secondary school	7 (16.7)
High School	12 (28.6)
Bachelor’s degree	1 (2.4)
Master’s degree	4 (9.5)
Working area, *n* (%)	
Craft industry	1 (2.4)
Business sector	3 (7.1)
Housewives	6 (14.3)
Industrial chemistry	3 (7.1)
Desk Jobs	15 (35.7)
Workers	6 (14.3)
Food service industry	1 (2.4)
Healthcare	2 (4.8)
Construction industry	1 (2.4)
Transport	1 (2.4)
More areas	3 (7.1)
Mean length of illness, *n* (%)	
About 5 years	15 (35.7)
6–14 years	18 (42.9)
More than 15 years	9 (21.4)
Exacerbations during the last year, *n* (%)	
None	20 (47.6)
1–3	17 (40.5)
More than 3	5 (11.9)
Hospitalisations last year, *n* (%)	
Less than 1	20 (47.6)
2	13 (31)
More than 2	9 (21.4)
Assistance during the last year, *n* (%)	
Yes	9 (21.4)
None	33 (78.6)
Type of assistance (if received), *n* (%)	
Senior Centre	0
Day care	1 (2.4)
Nurse	4 (9.5)
Physiotherapy	2 (4.8)
Other (i.e., clean)	2 (4.8)
Smoking Habits, *n* (%)	
Yes, active	4 (9.5)
No, never	7 (16.7)
Ex	30 (71.4)
Pack/year, *n* (%)	
Less than 10	4 (9.5)
10–20	14 (33.3)
More than 20	16 (38.1)
Alcohol habits, *n* (%)	
Never	21 (50)
Rarely	10 (23.8)
Sometimes	9 (21.4)
Quite often	0
Almost always	1 (2.4)
Always	0
Physical activity, *n* (%)	
Never	17 (40.5)
Rarely	8 (19)
Sometimes	8 (19)
Quite often	8 (19)
Almost always	0
Always	0
Medications, *n* (%)	
LABA	25 (59.5)
LAMA	25 (59.5)
CSI	28 (66.7)
Anxiolytics	14 (33.3)
Antidepressants	13 (31)
Number of medication, mean (SD)	9.2 (3.25)
Number of comorbidities, mean (SD)	2.88 (1.31)
BMI, mean (SD)	29.27 (7.64)

SD, standard deviation; Tot., total score; n, number of subjects; M, male; F, female; LABA, long-acting b2-agonists; LAMA, long-acting muscarinic antagonist; ICS, inhaled corticosteroids; BMI, Body Mass Index.

### 3.2. The arisen process underlying acceptance and adherence to NPPV in COPD

**Figure 1:**
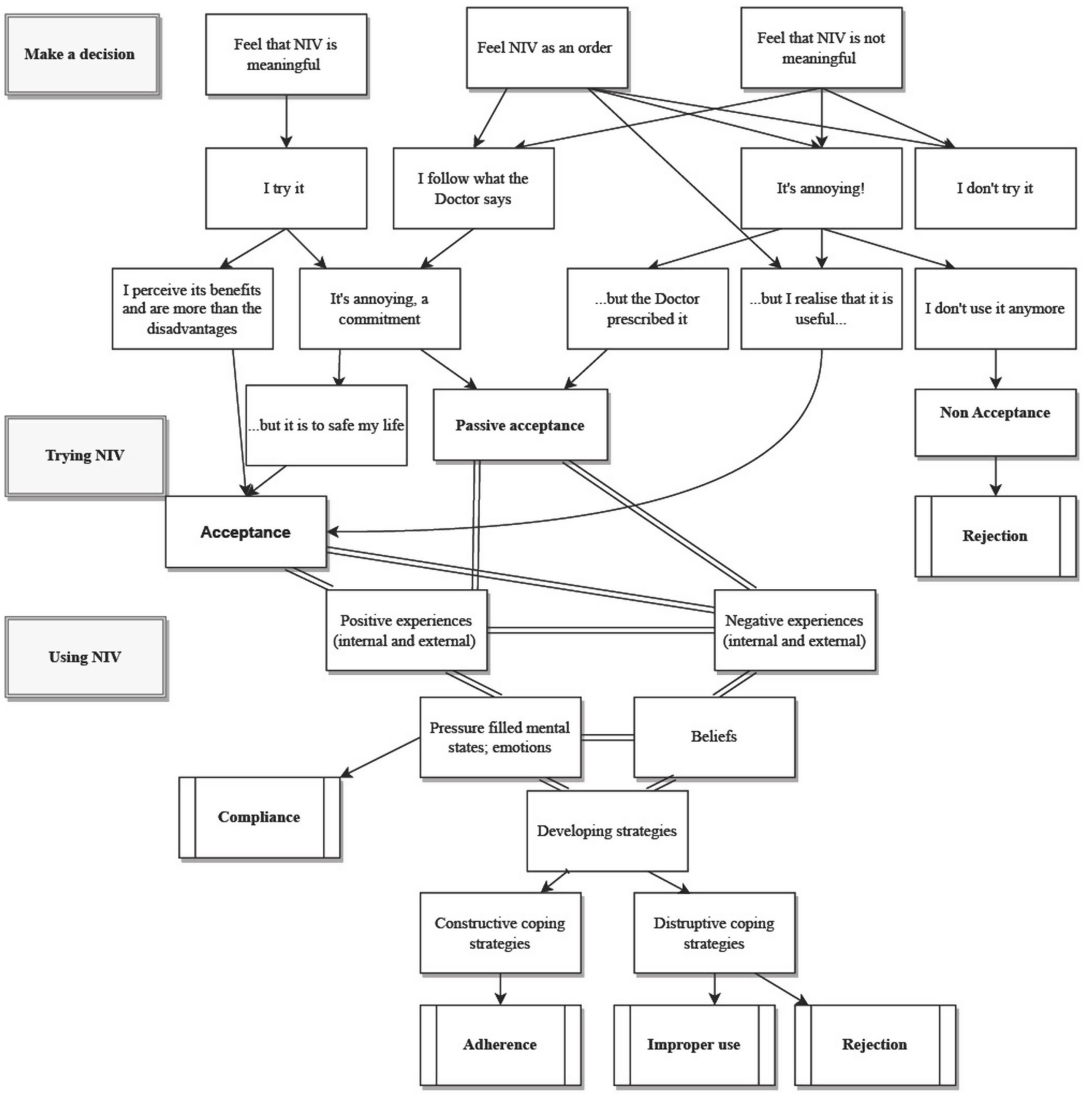
Patterns of acceptance and adherence to NPPV emerged from the analysis.

The process behind acceptance and adherence to NPPV in COPD people emerged as a theoretical model through which the participants tried to balance their clinical needs with the difficulties in approaching NPPV. The process is configured as dynamic, non-linear, and marked by positive and negative experiences, facilitators, and barriers as well as psychological features that can influence the process itself and the coping strategies that the participants implement to deal with the process itself. Indeed, COPD participants can develop adherence to the treatment when they adopt constructive strategies; while, if they do not make the effort to deal with the adaptation to NPPV or they manage them destructively, they can develop poor compliance, rejection, or improper usage, respectively (Figure 1).

Table 2 shows the 50 most used words during the adaptation process to NPPV in COPD.

**Table 2:** Overall word frequency of the 50 most words used during the process of adaptation to NPPV.

Word	Length	Count	Weighted percentage
Ventilation	12	182	0.22%
Interview	9	138	0.16%
Respect	8	125	0.15%
Deepen	13	104	0.12%
Illness	8	104	0.12%
Mask	8	102	0.12%
Overall	11	102	0.12%
Exercise	9	101	0.12%
Difficulty	10	90	0.11%
Route	8	90	0.11%
Refers	9	87	0.10%
Meeting	8	79	0.09%
Device	11	73	0.09%
Problems	8	72	0.09%
Respiratory	12	71	0.08%
Something	8	69	0.08%
Reflect	10	69	0.08%
Could	8	68	0.08%
Let us do	8	65	0.08%
Machine	8	65	0.08%
Nuisance	8	63	0.07%
Possible	9	63	0.07%
The oxygen	10	61	0.07%
Elaborate on	12	59	0.07%
Relaxation	12	59	0.07%
Tells	8	56	0.07%
Salute	9	55	0.06%
Pathology	9	53	0.06%
To take	8	53	0.06%
May be possible	9	52	0.06%
Adds	8	51	0.06%
Hospital	8	51	0.06%
We reflect	11	51	0.06%
Worried	11	49	0.06%
Invasive	8	48	0.06%
To walk	9	46	0.05%
Possibility	11	45	0.05%
Recovery	8	45	0.05%
Medicines	8	44	0.05%
Showing	11	43	0.05%
Explore	10	42	0.05%
Family	8	42	0.05%
To breath	9	42	0.05%
Strategies	9	42	0.05%
Physiotherapy	12	41	0.05%
Management	8	39	0.05%
Try	9	38	0.04%
Mindfulness	11	36	0.04%
Hard	9	35	0.04%
Concern	14	35	0.04%

### 3.3. Patterns of adaptation and adherence to NPPV

The first step that characterized all the patients interviewed is represented by deciding on accepting the idea to try or reject NPPV when the physician proposes it [i.e., *“a huge new breathing machine that looks like a harness”* (A0103)]. An essential component of this first phase is a good alliance between the pulmonologist and the patient. Moreover, feelings of meaningfulness [i.e., *“I never had it and I do not understand why I should wear it”* (B0205)], perceived orders [i.e., *“I feel compelled, obliged. They are all with rifles pointed”* (B0202)], or hope of success of NPPV [i.e., *“It cannot do miracles, but I hope it helps me getting better”* (B0203)] were significantly related to the decision of accepting or rejecting NPPV at first sight.

Some COPD patients perceived NPPV as a command and did not completely understand the relevance and the objective of the device or, on the contrary, they did not recognize it as significant for themselves and their wellbeing. Therefore, most people totally and immediately rejected NPPV, yielding to the fear of losing autonomy as well as the preservation of self-determination, especially if they had few symptoms or lacked knowledge about their respiratory condition. On the other hand, some COPD patients considered NPPV as probably meaningful to improve the therapeutic and routine care processes, both witting of their respiratory conditions and fearing their deterioration. Other participants underlined their conception of the decision to undertake NPPV as resting solely with the doctor. In these cases, they immediately took a position against NPPV or decided to passively follow the physician’s indications. Before perpetrating NPPV’s usage, some COPD participants suspended it after trying it a few times and/or persevering on the basis of its everyday effects on their respiratory conditions. Indeed, some people accepted it simply because of the medical prescription or persisted in using NPPV despite feeling it was a burden.

Most of the involved patients experienced both positive (79 references) and negative feelings (140 references) related to NPPV’s use. Examples of *internal positive experiences* were embodied by the benefits perceived during both daily life and night rest, while the *external positive experiences* could be represented by the perceived support from loved ones or the healthcare professionals.

On the other hand, *internal negative experiences* were depicted by the scarcity of a substantial effect on the respiratory symptoms or negative feelings such as fear of losing independence or of having to use NPPV forever. *External negative experiences*, for instance, were portrayed by a lack of professional support or listening and clarifications by healthcare professionals.

Both positive and negative experiences are provided to raise or strengthen the beliefs that often play a relevant role in the decision-making process to begin NPPV [i.e., *“I cannot decide, the decision is in the doctor’s hands”* (Z02527)]. The experiences also contributed to evoking emotions [i.e., *“That machine ruins my life, and having to use it worries me. It makes me feel anxious, that they have already diagnosed me in the past.”* (BO202)] and allowed us to develop pressure-filled mental states [i.e., *“My granddaughter does not want to see me with that mask. She is 32 years old and lives together with her boyfriend and she often visits me. I do not take it kindly.”* (AO104)].

Both internal and external, positive, and negative experiences as well as beliefs and emotions and stressful mind states actively and dynamically interact with each other, that in turn interface with acceptance and adherence and the coping strategies developed by the patients to face experiences and emotions. On the other hand, some COPD participants did not develop constructive coping strategies, resulting in compliance with NPPV. In this context, the constructive coping strategies favored the flourishing of adherence, while the destructive coping strategies opened the doors to improper use or a total rejection of the NPPV (Figure 1).

### 3.4. Coping strategies

The analysis of coping strategies resulted in the creation of two categories: *consolidated strategies* (*n* = 16, 38.9%) and *new strategies* (*n* = 21, 50%). Both of them allow us to distinguish between “constructive” and “destructive” coping strategies, which represent a form of adaptive or maladaptive coping, respectively (Lazarus and Folkman, 1984). Five participants (11.9%) recurred to both consolidated and new strategies. The most frequently used constructive coping strategies were represented by: adaptation, which consists of gradually increasing the amount of NPPV practiced according to the prescribed indication of the pulmonologist (*n* = 5; 11.90%); intellectualization, paying more attention to the facts than to the emotions (*n* = 2; 4.76%); irony or sense of humor (*n* = 3; 7.14%); performing NPPV as a ritual (*n* = 6; 14.28%); fantasy, which allowed access to a world of possibilities (*n* = 1; 2.38%); and distraction, opting for hobbies (*n* = 9; 21.42%).

On the other hand, the most common destructive coping strategies were depicted by: avoidance, which consisted of not asking for more information about the clinical conditions, fearing a deterioration, not asking for help, fearing becoming a burden or losing autonomy (*n* = 2; 4.76%; denial *n* = 4; 9.52%); rationalization, that is, looking for reasons to stop using NPPV (*n* = 5; 11.90%); and procrastination, which comprised of postponing bedtime to reduce the hours of NPPV practice (*n* = 4; 9.52%) (Table 3).

**Table 3:** Examples of constructive and deconstructive coping strategies and the respective number of participants who referred to each strategy.

	Constructive	Examples	Deconstructive	Examples
Coping strategies				
	Adaptation (*n* = 5; 11.90%)	<<*I could try to use the ventilation a few hours at a time, increasing them more and more...> >* (H0808)	Avoidance (*n* = 2; 4.76%)	<<*At home, nobody helps me, I do not want to be helped. I’m against it, I’ve always done it for everyone*> > (AO103)
	Intellectualization (*n* = 3; 7.14%)	<<*Today, for example, with the bike I did better and calmed down. I put in the ventilation and kept it all over the night*>>(E0508)	Denial (*n* = 4; 9.52%)	<<*I do not think it depends on the treatments I’m doing, but that’s why it is, I’m old now*> > (AO102)
	Performing rituals (*n* = 6; 14.28%)	<<*It’s like taking pills, you’ll use to do it*>> (AO102)	Rationalisation (*n* = 5; 11.90%)	<<*For now, I do not feel a real benefit,* “(BO203)*”*; *Let us say I think I might be without it. I’ve been up to date ... If I need to do it>>* (AO102)
	Fantasy (*n* = 1; 2.38%)	<<*I think there is something magical. Maybe it’s Dr. XX! The soup ... who knows!*> > (Y02425)	Procrastination (*n* = 4; 9.52%)	<<*I’ll go to bed as late as possible, to reduce the ventilation hours ... it gives me too much trouble!> >* (BO204)
	Irony (*n* = 3; 7.14%)	<<*It will continue, but you must ironize*> > (Z02527)		
	Distraction (*n* = 9; 21.42%)	<<*I enjoy crosswords, solitude, deep breathing exercises, and prayer. At 74 I cannot change my life*> > (X2323)		

It is also important to note that some of these coping strategies were combined and were about a change of perspective about COPD or NPPV (constructive), as well as a return to the past, which hinders both awareness and changing perspective (destructive).

Adherence to NPPV may vary among individuals and may be influenced by various factors. From our study, it appears that the presence of a caregiver can certainly play a role in supporting and encouraging treatment adherence. However, it is important to note that adherence does not depend solely on the presence of a caregiver. Some factors that may affect adherence to NPPV in a person with COPD and that emerged from the study are shown in Table 4.

**Table 4:** Factors that may affect adherence to NPPV in COPD as emerged by the analysis.

Factor	Description	Citation
Education and understanding (*n* = 36; 85.71%; 40 references, 12.97% coverage)	A good understanding of the advantages and appropriate application of NPPV is essential. The patient must comprehend how the medication works and why it is crucial for controlling the symptoms of COPD. Health care professionals are required to give precise instructions and address any worries or inquiries.	*<< They put a machine on me at night. But it’s too much to keep it all night. I’ve never had it and I do not understand why I should put it on and then I get short of air with the mask, it suffocates me and they do not understand it…> >* (B0205)
Comfort and fit (*n* = 5; 11.90%; 5 references, 1.1% coverage)	Comfort and fit of the NPPV equipment, such as the mask or nasal prongs, can have a big impact on adherence. Equipment fitting, adjusting, and review on a regular basis can reduce discomfort and increase compliance.	*<< At first the mask bothered me. Especially the olives. Now, with the new one, it’s better. The last one is more comfortable > >* (C0507)
Side effects (*n* = 30; 71.42%; 56 references,13.47% coverage)	Some people may have NPPV-related side effects or discomfort, including as dry nasal passages, itchy skin, or claustrophobia. To increase adherence, health care practitioners must swiftly address these issues.	*<< My skin is delicate and that mask is a problem, I would like one that does not scar my face. All the skin on my face is damaged> >* (B0202)
Self-motivation (*n* = 10; 23.8%; 11 references, 5.16% coverage)	Adherence to NPPV frequently necessitates self-motivation and self-mastery. Even when there is no caregiver present, it is crucial that people commit to utilize therapy as directed and acknowledge its advantages.	*<<Yesterday with Dr XX we looked at the ventilator...if I have to bring it, I’ll bring it. It’s up to the doctor to give the device...experiences are many, of all colors> >* (G0710)
Support network or caregiver presence (*n* = 21, 50%; 34 references,24.9% coverage)	Although the absence of a devoted caregiver may make therapy compliance more challenging, individuals may turn to other sources of support, such as family, friends, or support groups. However, they are often afraid of being a burden to the latter. People can maintain their motivation and accountability with social support.	*<<My son is young, 21 years old, and he is out of the house all day because he works…> >* (Z2527)

## 4. Discussion and conclusion

### 4.1. Discussion

The findings based on COPD participants’ adaptation to NPPV pointed to both strengths and difficulties of the process, which has been characterized to be non-linear and dynamic. Participants reported the NPPV intervention as both a burden and a relief, an aspect that emerged along all the three principal identified phases: making a decision, trying NPPV, and using NPPV. According to previous studies, the adaption to NPPV could be defined as a lengthy and progressive process, a gradual familiarization, by which COPD patients try to modify their routine healthcare to introduce a new device (Lindahl et al., 2005; Ngandu et al., 2016). In this respect, it is important to note that this study paid attention to the adaptation to NPPV in CRF, because as previous studies noted, several patients in ARF described that experience as confused, not remembering all from the acute stage (Torheim and Gjengedal, 2010). On the other hand, Sørensen’s theoretical model, which paid attention to the patients admitted to the hospital because of ARF, observed similar behavioral patterns: indeed, in both studies, an early failure in approaching NPPV was associated with a lack of tolerance and/or discontinuing NPPV until a complete dismission. Moreover, the authors identified the meaningfulness associated with the participants’ experiences as a relevant strength, which could be led back to the constructive coping strategies that emerged from our model (Sørensen et al., 2014). Our study draws attention also to a peculiar vulnerability to failure (Duan et al., 2019) at both the beginning of the NPPV treatment and after a period of successful adaptation (Carratu et al., 2005; Sørensen et al., 2014). The participants’ process of adaptation allowed us to note some individual variations between those who immediately accepted NPPV and those who became just compliant with it, those who accepted it after a long time but developed adherence, and those who completely refused it. These variations were related to both the inner and external, positive, and negative experiences related to the device as well as to the beliefs, emotions, and mental states felt. Unsolved breathlessness and the implemented coping strategies, contrary to the case of ARF (Sørensen et al., 2014), seemed to play an important role in improving or decreasing the acceptance and then adherence to NPPV.

The study also underlined the relevance of the positive support received from both the family members and the healthcare professionals during the adaptation to the device, confirming that the participants believed themselves inbetween dependence and autonomy at various levels because of NPPV. Indeed, their need for support emphasized the relevance of relationships as means for developing constructive coping strategies (Torheim and Gjengedal, 2010; Kvangarsnes et al., 2013; Torheim and Kvangarsnes, 2014). It must be underlined that adherence is a complicated subject and that every person’s situation is unique. It is fundamental to remind patients to consult a medical expert who can offer customized advice and assistance if there are some troubles adhering to NPPV or any other medical treatment. On the other hand, it is essential to take the ethical ramifications of top-down decision-making without the patient’s point of view into account. The introduction of NPPV can have psychosocial and emotional implications for patients, as it may disrupt their daily routines, limit mobility, or cause discomfort. Considering these aspects, together with the person’s values, building trust in the treatment process, and the centrality of patient wellbeing from the very beginning of the decision to embark on the NPPV adjustment pathway, can serve as carriers to encourage one’s active involvement in the treatment process (Rapelli et al., 2023). Healthcare personnel should participate in shared decision-making procedures that put the patient’s autonomy, informed consent, trust, and personal values and objectives first. Healthcare practitioners can improve patient participation and encourage more moral and patient-centered care by considering the patient’s perspective. The results of our study are in line with those found in the literature, which encourages the consideration of these aspects in the engagement process, even in populations with chronic respiratory diseases in adaptation to external devices (Rapelli et al., 2022).

Our findings fit the data from empirical situations, abstaining from adopting predetermined theories. Recruitment was limited to people with moderate or severe COPD in need of NPPV: therefore, we cannot assume that our findings reflect all the possible details for the entire range of patterns of adaptation to NPPV. This study was conducted in a single rehabilitation ward, so the adaptation to NPPV and the management of COPD patients might reflect the local culture. In this case, we defined the sampling as purposive because we started by selecting only those patients who had a confirmed diagnosis of COPD according to the Global Initiative for Chronic Obstructive Lung Disease (GOLD) criteria, from moderate [Stage 2-50% ≤ Forced Expiratory Volume in 1 Second (FEV_1_) < 80% predicted] to severe (Stage 3- 30% ≤ FEV_1_ < 50% predicted) along with the use of NPPV and without cognitive impairment. Since this was a specific sample, purposive sampling allowed us to include individuals with unique insights or experiences that may contribute to a comprehensive understanding of the phenomenon under investigation. Subsequently, sampling was conducted based on the themes that progressively emerged. In the future, it might be useful to consider different rehabilitation centers or even different countries, as health professionals and/or different approaches to NPPV might differ. Subsequent quantitative studies may help in understanding which of the factors identified as influencing, from the patient’s point of view, the process of adaptation to NPPV may influence to a greater or lesser extent (e.g., whether being without support, as in the case of one of our participants, significantly affects adherence to NPPV). Similarly, it would be very interesting to know whether having difficulty in changing certain lifestyle habits (e.g., smoking cessation) correlates significantly with difficulty in terms of adaptation to NPPV, an aspect that in our study was found to be related to motivation based on what participants reported, but which could not be verified. Nevertheless, the data included a considerable variation in COPD patients’ experiences, length of the adaptation process to NPPV, comorbidities, age, and gender. Counseling interviews allowed us to gain insight into the participants’ adaptation to NPPV, paying attention to their beliefs, thoughts, and behaviors, and following the adaptations to NPPV step by step. This approach allowed us to comprehend a limited research area, even if the sample of people with COPD involved in our study is relatively small and they were all psychologically supported during the NPPV’s adaptation.

### 4.2. Conclusion

For COPD individuals, using NPPV represents both a necessity and a stressful experience at the same time. The patterns of adaptation arising from our study underlined the importance of an interrelationship between beliefs, state of mind, emotions, behaviors, and coping strategies, as a fundamental matching to improve or decrease acceptance and then, adherence to the device. These findings offer clinicians and policymakers a series of patients’ perspectives, paying attention to the psychological factors underlined by the adaptation to NPPV, discussing both constraints and strengths, that could be useful to manage a new model of care related to the introduction of a medical device in the patient’s routine.

### 4.3. Practice implications

Further studies that test this theoretical model in other countries as well as involving people with very severe COPD are needed. Moreover, our study highlighted the importance of the caregiver’s role during the adaptation process to NPPV, suggesting how considering the relationship between caregiver and patient could be relevant. Supportive material might help visualize complex information. Finally, translating the model into clinical practice, considering also other vulnerable patients who need NPPV, can help to gain insight into the better way of delivering its usage and management.

## Data availability statement

The data that support the findings of this study are available from the corresponding author, [EV], upon reasonable request.

## Ethics statement

The studies involving human participants were reviewed and approved by Ethics Committee of the IRCCS Fondazione Don Carlo Gnocchi (reference: 15th February 2015), in Milan (Italy), in the mainframe of a large RCT on acceptance and adherence to NIV in people with COPD (ref. ClinicalTrials.gov ID: NCT02499653). The patients/participants provided their written informed consent to participate in this study.

## Author contributions

EV contributed to the design and implementation of the research, the analysis of the results, and the writing of the manuscript. PB and FP authors provided critical feedback and validated the analysis. FP supervised the entire work. All authors contributed to the article and approved the submitted version.

## Conflict of interest

The authors declare that the research was conducted in the absence of any commercial or financial relationships that could be construed as a potential conflict of interest.

## Publisher’s note

All claims expressed in this article are solely those of the authors and do not necessarily represent those of their affiliated organizations, or those of the publisher, the editors and the reviewers. Any product that may be evaluated in this article, or claim that may be made by its manufacturer, is not guaranteed or endorsed by the publisher.

## Abbreviations

LTOT: long-term oxygen therapy
NPPV: non-invasive positive pressure ventilation
ARF: acute respiratory failure
CRF: chronic respiratory failure
COPD: chronic obstructive pulmonary disease
GOLD: global initiative for chronic obstructive lung disease
RCT: randomized controlled trial
FEV_1_: forced expiratory volume in 1 second
MMSE: mini-mental state examination
CGT: constructivist grounded theory
COREQ: consolidated criteria for reporting qualitative research
